# Innate immunology in COVID-19—a living review. Part II: dysregulated inflammation drives immunopathology

**DOI:** 10.1093/oxfimm/iqaa005

**Published:** 2020-12-08

**Authors:** Patrícia R S Rodrigues, Aljawharah Alrubayyi, Ellie Pring, Valentina M T Bart, Ruth Jones, Clarissa Coveney, Fangfang Lu, Michael Tellier, Shayda Maleki-Toyserkani, Felix C Richter, D Oliver Scourfield, Ester Gea-Mallorquí, Luke C Davies

**Affiliations:** 1 Division of Infection and Immunity, School of Medicine, Systems Immunity Research Institute, Cardiff University, Cardiff, UK; 2 Nuffield Department of Medicine, Viral Immunology Unit, University of Oxford, Oxford, UK; 3 Dementia Research Institute, Division of Infection and Immunity, School of Medicine, Cardiff University, Cardiff, UK; 4 Kennedy Institute of Rheumatology, University of Oxford, Oxford, UK; 5 Sir William Dunn School of Pathology, University of Oxford, Oxford, UK

**Keywords:** Innate immunology, COVID-19, Neutrophils, Monocytes, NK cells, Macrophages

## Abstract

The current pandemic of coronavirus disease 2019 (COVID-19) caused by severe acute respiratory syndrome coronavirus 2 (SARS-CoV-2) poses a global health crisis and will likely continue to impact public health for years. As the effectiveness of the innate immune response is crucial to patient outcome, huge efforts have been made to understand how dysregulated immune responses may contribute to disease progression. Here we have reviewed current knowledge of cellular innate immune responses to SARS-CoV-2 infection, highlighting areas for further investigation and suggesting potential strategies for intervention. We conclude that in severe COVID-19 initial innate responses, primarily type I interferon, are suppressed or sabotaged which results in an early interleukin (IL)-6, IL-10 and IL-1β-enhanced hyperinflammation. This inflammatory environment is driven by aberrant function of innate immune cells: monocytes, macrophages and natural killer cells dispersing viral pathogen-associated molecular patterns and damage-associated molecular patterns into tissues. This results in primarily neutrophil-driven pathology including fibrosis that causes acute respiratory distress syndrome. Activated leukocytes and neutrophil extracellular traps also promote immunothrombotic clots that embed into the lungs and kidneys of severe COVID-19 patients, are worsened by immobility in the intensive care unit and are perhaps responsible for the high mortality. Therefore, treatments that target inflammation and coagulation are promising strategies for reducing mortality in COVID-19.

## Graphical Abstract

**Graphical Abstract:**
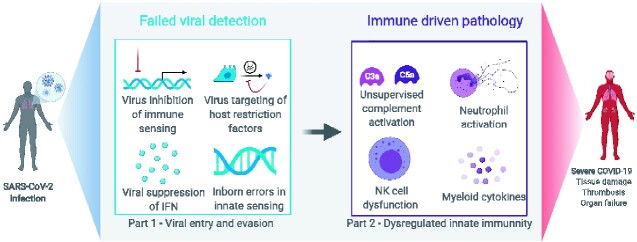

**Highlights**

- NK cells are thought to undergo mass migration to the lungs, depleting circulating populations and may contribute to tissue damage
- Complement activation is linked to virus removal, but is implicated in neutrophil activation and thrombosis
- A robust early macrophage IFN-response is associated with mild disease, while high levels of the cytokines IL-6, IL-10 and IL-1β in the early stages of COVID-19 are linked to poor outcome
- Neutrophil activation causes tissue damage and an increased risk of potentially fatal immunothrombotic events in the lungs and kidneys of COVID-19 patients
- Monocyte HLA-DR expression, neutrophil activation and the ratio of neutrophils to lymphocytes might be a potential identifier of high-risk patients

**Box 1: Why does your reviewed topic matter in the pandemic?**

The first responders to viral infection in coronavirus disease 2019 (COVID-19) are the cells of the innate immune system, including natural killer (NK) cells, monocytes, macrophages and neutrophils. After migrating to the site of infection and recognizing the virus from conserved features, innate cells initiate responses to eliminate the virus and coordinate the adaptive immune system. However, in some cases, innate cells have been shown to contribute to viral dissemination and tissue damage which is associated with severe COVID-19 disease and fatal outcomes. Therefore, it is critical to evaluate innate immunity in the context of COVID-19 to predict outcome and facilitate development of therapeutic interventions.

**Box 2: What is the consensus?**

A hallmark of life-threatening COVID-19 is a pathological hyperactive immune cell response brought about by the inability to control virus. NK cells that are lost in the circulation traffic to the lungs where they may contribute to inflammatory damage. Alveolar macrophages and monocytes are implicated in cytokine propagation. Macrophage virus infection and activation may inhibit their ability to cloak inflammation. Non-classical monocytes and neutrophils are increased in the blood of severe COVID-19 patients suggesting high bone marrow turnover. A cytokine storm has also been reported in COVID-19. Though high cytokine levels such as interleukin (IL)-6, IL-10 and IL1-β are linked to disease severity, these cytokine elevations are thought to only occur early in the disease followed by reported immunosuppression. Complement, damage and cytokines are implicated in excessive neutrophil activation, triggering fibrosis and damaging thrombotic events, thought to be the key drivers of mortality in COVID-19. As such, a high ratio of neutrophils to lymphocytes (including NK cells and T cells) and high D-dimer levels can predict COVID-19 disease outcome.

## INTRODUCTION

Severe acute respiratory syndrome coronavirus 2 (SARS-CoV-2) is a novel betacoronavirus causing coronavirus disease 2019 (COVID-19). COVID-19 has had devastating effects on human health and social and economic aspects worldwide. SARS-CoV-2 is highly infectious and while a proportion of patients present with asymptomatic disease, clinical manifestations can include fever, cough, loss of smell/taste, fatigue, difficulty breathing and organ dysfunction [acute respiratory distress syndrome (ARDS)], which for a small percentage of patients will be fatal. The risk of developing severe disease is associated with old age, gender, ethnicity and underlying health conditions including diabetes, obesity and heart disease. Severe outcomes likely involve a defective viral sensing and a dysregulated immune response. Part 1 of this review discussed the mechanisms of SARS-CoV-2 host cell entry and immune evasion. Part 2 assesses the current understanding of how innate immune responders [natural killer cells (NK), myeloid cells and neutrophils] contribute to pathogenesis.

The innate immune system provides the first line of defence against infection, rapidly respond to pathogens through recognition of conserved features, but are also important for adaptive immune responses. However, such lack of specificity can contribute to tissue damage and undermine adaptive immunity, thus a fine balance is required to protect the host.

### The complement system and ‘cytokine storms’ in COVID-19

The complement system comprises several plasma proteins that opsonize or lyse pathogens and orchestrate host defence [1–3]. Dysregulated activation of complement plays a role in chronic inflammation, endothelial cell dysfunction, intravascular coagulation and thrombus formation eventually leading to multiple organ dysfunction syndromes and death [4], and has been implicated in the pathophysiology of Middle East respiratory syndrome, SARS [5–8] and COVID-19 [5]. Immunohistochemistry analysis of lung tissue from patients who died of COVID-19 revealed deposition of complement components mannose-binding lectin, C4, C3 and the terminal membrane attack complex C5b-9 in alveolar epithelial/inflammatory cells and alveolar spaces [9]. Additionally, elevated plasma C5a and sC5b-9 have been observed in patients with moderate and severe COVID-19 [10]. C5a is a potent signalling molecule that activates immune cells to release cytokines, including tumour necrosis factor (TNF), interleukin (IL)‐1β, IL‐6 and IL‐8 within hours of infection [11]. Despite some suggestive evidence, the complement system has received little attention in the search for effective anti-inflammatory treatment strategies despite multiple intuitive targets in COVID-19 [12], listed in Table 1.

**Table 1:** Potential treatments

Therapy	Target	Mechanism	Reference
Monalizumab	NKG2A	As upregulation of NKG2A is associated with reduced lymphocyte to neutrophil balance, inhibition of NKG2A may improve lymphocyte numbers and therefore virus control.	van Hall *et al. [13]* Antonioli *et al. [14]*
Sarilumab and Tocilizumab	IL-6	Higher blood concentrations of IL-6 were reported to be predictive of fatal outcome in COVID-19 patients therefore blocking IL-6 using antibodies may be effective.	Ruan *et al. [15]* Guaraldi *et al. [16]* Tomasiewicz *et al. [17]* ClinicalTrials.gov Identifier: NCT04320615 (no statistically significant differences in ventilator-free days between the drug and placebo)
Adalimumab	TNF	Proven to reduce inflammation in many diseases and thus promising for severe COVID-19	Mahase [18]
Anakinra	IL-1 receptor (agonist)	Competitively inhibits IL-1 binding to the IL-1 type I receptor. Increased IL-1 concentrations have been reported in COVID-19 patients. IL-1a and IL-1b have been implicated in severe COVID-19 disease.	Giamarellos-Bourboulis *et al. [19]* Huang C *et al.* [20] Ong *et al.* [21] Huet *et al.* [22] Cavalli *et al. [23]*
IFN I and IFN II targeting drugs	IFN blocking	Late IFN responses are associated with hyperinflammation in severe COVID-19 disease. Due to potential off-target effects, blocking IFN I responses may be more effective than blocking IFN II responses.	Hemann *et al. [24]* Sallard *et al. [25]* Prokunina-Olsson *et al. [26]*
IFN I supplementation	IFN-β, IFN-α2b, IFN-α1b	Severe COVID-19 patients have shown reduced IFN I responses. IFN I supplementation reduced the duration of inflammatory markers in mild disease and prevented COVID-19 infection in highly exposed individuals.	Hung *et al. [27]* Zhou *et al. [28]* Meng *et al. [29]* (prevented infection in highly exposed individuals)
Baricitinib, Ruxolitinib	JAK 1 and JAK 2	May prevent virus entry into cells as well as beneficial anti-inflammatory activity. Inhibits NK cell activity, DC development and function which could suppress antigen-specific T cell responses.	Stebbing *et al. [30]* Elli *et al. [31]*
Gimsilumab, mavrilimumab, Sargramostim (Human recombinant GM-CSF)	GM-CSF replacement	GM-CSF promotes proinflammatory responses. GM-CSF expression increases T helper 1 cells and monocytes in COVID-19 patients, particularly those in intensive care. GM-CSF also plays a role in alveolar macrophage physiology and might protect against viral related injury in early stages.	Zhou *et al. [32]* De Luca *et al. [33]* Clinicaltrials.gov identifier: NCT 04326920
Monoclonal antibodies	Immune checkpoint blockade e.g. PD-1, NKG2A and CD39.	Immune checkpoint therapy, approved for melanoma, has been shown to upregulate effective immune responses by blocking inhibitory markers on immune cells and infected cells. In COVID-19 disease it was shown that NK cell responses could be enhanced using immune checkpoint blockade.	Demaria *et al. [34]*
Dexamethasone	Glucocorticoid receptor	Increases anti-inflammatory genes (e.g. IκB-α), reduces pro-inflammatory genes (e.g. COX2)	Johnson and Vinetz [35]
Hydrocortisone	Glucocorticoid receptor	Increases anti-inflammatory genes (e.g. IκB-α), reduces pro-inflammatory genes (e.g. COX2)	Mahase [36]
Anti-coagulants	Coagulation cascade	E.g. Warfarin, apixaban, betrixaban, dabigatran, edoxaban and rivaroxaban	Nadkarni *et al.* [37]
Ecluzimab	C5a	Inactivates C5a, removing anaphylatoxin activity	Cugno *et al.* [10] Campbell and Kahwash [12]
High-dose IV immunoglobulin	C3b	Sequesters C3b stops both MAC activity and C5a production	Cugno *et al.* [10]

In addition to complement, pathogen-associated molecular patterns (PAMPs) and damage-associated molecular patterns (DAMPs) can also induce cytokine release. Cytokines play an important role in promoting immunopathology and immune dysfunction: ‘Cytokine storms’ reported in severe SARS-CoV-2 infected patients (reviewed in detail by Ragab *et al.* [38] refers to an elevated and dysregulated release of pro-inflammatory cytokines and chemokines, including IL-6, TNF-α, IL-1, interferon (IFN)-γ and MCP-1. Some studies report only modest cytokine elevations [39], particularly early in disease, while other studies correlate IL-6 and TNF-α levels with disease severity [40–43] and are therefore promising targets for treatment (Table 1). However, there is no consensus on reported cytokine levels, particularly IL-6 (approximate range = 10pg/mL to >20 ng/mL), which could be attributed to analytical method or infection time-point. Indeed, it has been suggested that early IL-6 elevations are followed by cytokine immunosuppression [39]. Cytokines related to tissue repair (e.g. IL-10, IL-4 and IL-5) are also upregulated in SARS-CoV-2 infection [20]. Therefore, a ‘cytokine storm’ driving COVID-19 pathology is controversial and some studies reject this terminology [39, 44]. Yet, the inflammatory cytokine response remains important in COVID-19 as severe patients often display symptoms usually associated with sepsis [45]. Here we address the role of innate cells in this response.

### NK cell dysregulation in COVID-19

NK cells are a heterogeneous subset of innate lymphocytes that play a key role in early anti-viral host defence [46, 47]. NK cells directly recognize virus-infected cells, lyse them and facilitate crosstalk between innate and adaptive immune responses [48]. Their activation is regulated by receptors that sense cell stress or viral-specific signatures and finely control the balance between activation and inhibition. In COVID-19 pathology, a functional NK response is associated with enhanced SARS-CoV-2 neutralization by antibody-dependent cellular cytotoxicity (ADCC) [49], increased numbers of the cytokine-producing Cluster of Differentiation (CD)56^bright^CD16^-^ NK cell subset and upregulated *IFN-* and *XCL2-*related genes [50]. Therefore, an effective NK cell response is likely crucial to control COVID-19 disease. Without the support of healthy phagocytes ADCC may increase inflammation (e.g. through DAMP release), however, reports of this and unassisted NK cell lysis of virally infected cells are mostly absent in COVID-19 [51], including peptide-loaded MHC-1 presentation and recognition. SARS-CoV-2 ORF8-mediated downregulation of MHC-I in infected cells [52] could also either promote or inhibit NK cell-mediated lysis, though this is also not known in COVID-19.

Some studies suggest a preferential reduction in circulating NK cells and reduced NK functionality are associated with severe COVID-19 [34, 53–55] disease but it is unclear whether this is due to cell trafficking to infected organs or NK cell death. Transcriptomic analysis suggested increased apoptosis-related pathways in circulating lymphocytes from COVID-19 patients [56]. Conversely, a recent scRNAseq analysis suggested increased NK cell representation in the lungs of COVID-19 patients [56] and genes involved in cell trafficking were enriched in bronchoalveolar lavage (BAL) NK cells from severe COVID-19 patients [57]. BAL fluids from COVID-19 patients also contained more chemokines which potentially could increase NK cell recruitment [58]. Though the mechanism has yet to be elucidated, CXCR3 ligand-producing cells are increased in COVID-19 patient’s lungs [58], which may indicate a CXCR3-dependent mechanism for NK cells recruitment to the lung as described in influenza A infection [59]. NK cells in the periphery may migrate to the lung and may contribute to immune-mediated lung damage in COVID-19 though this is not yet understood.

SARS-CoV-2 infection also modulates the NK cell phenotype causing functional changes that could affect patient outcome. Recent data from COVID-19 patients with pneumonia or ARDS suggest an increase in NK cell exhaustion, characterized by elevated NKG2A, PD-1 and CD39 expression in patients with severe infection [34]. Targeting these molecules has been suggested as therapeutic strategy for COVID-19 (Table 1). In keeping with an exhaustion phenotype, NK cells in COVID-19 patients are functionally impaired [53, 60], which could be explained by hyperinflammation, particularly the persistent elevation of IL-6 in COVID-19 patients [60, 61]. In contrast, ‘adaptive’ NK subpopulations with enhanced ADCC responses, actively proliferated and expanded in the periphery of severe COVID-19 patients seropositive for cytomegalovirus (CMV) [57]. This expansion did not correlate with CMV IgG, suggesting these cells could be repurposed towards combating SARS-CoV-2. Consistent with this, HLA-E, a major ligand for NKG2C-expressing adaptive NK cells, is upregulated in BAL fluid of COVID-19 patients [57]. Additionally, increased prevalence of the NKG2C^del^ variant, associated with reduced NKG2C expression, has been found in severe COVID-19 which may highlight the importance of the NKG2C–HLA-E axis on antiviral activity [62].

In severe disease, CD56^bright^ NK cells, (enhanced in mild cases and usually associated with cytokine release/immunoregulation) exhibited higher levels of cytotoxic mediators [57], suggesting that NK cell regulation is abandoned for cytotoxicity, perhaps further promoting tissue damage. However, overall, while NK cell dysregulation has been described in SARS-CoV-2 patients there is still lack of understanding of early NK cell-mediated responses and the balance between pathological versus protective NK cell responses at the sites of infection.

### Monocytes and macrophages contribute to dysregulated inflammation

Macrophages, monocytes, and dendritic cells (DCs) are sentinels of the immune system that express a broad repertoire of PAMP, DAMP and complement receptors. They also present antigen to trigger adaptive immune responses and are equipped with antibody Fc receptors to be effector cells. Under physiological conditions, macrophages are tissue accessory cells that dampen inflammation via scavenging of dead cells and DAMPs such as ATP [63]. Tissue-resident macrophages are likely the first to encounter SARS-CoV-2 in the lung: alveolar macrophages can detect viral components via PRRs and produce Type I IFNs (IFNs I; IFN-α and IFN-β): essential cytokines in the antiviral immune response [64]. These cells will also recruit other immune cells to trigger inflammation [65], which overrides their usual anti-inflammatory function, as the ability of macrophages to cloak inflammation is ineffective when the tissue damage is considerable [63]. Reports of two fatal COVID-19 cases showed increased macrophage recruitment in alveolar cavities expressing high levels of CD68, indicating an activated state [66]. Those macrophages were shown to secrete moderate amounts of pro-inflammatory IL-6 and TNF-α but high levels of anti-inflammatory IL-10.

As macrophages express ACE-2, direct infection with SARS-CoV-2 could prevent IFN I production (a viral evasion mechanism discussed in Part 1 of this review) impairing viral control and causing hyperinflammation [67]. Mild SARS-CoV-2 infection has been associated with robust IFN I responses [68] which might account for the higher proportion of non-severe cases of COVID-19 compared to SARS-CoV. However, defective IFN I responses have been reported in severe COVID-19 cases, where patients lacking IFN-α production had poorer outcomes [69, 70]. Murine SARS-CoV studies suggest early IFNs I support clearance of virus, while delayed or absent responses cause elevated cytokine levels and impaired adaptive immune responses [67]. Consequently, blocking IFN I at late stages of the disease may be advantageous (Table 1). While alveolar macrophages can produce IFN I, they are not the primary source during viral infection. Therefore, viral downregulation of IFN I in macrophages unlikely drives the dampened immune response and macrophages will not be the primary source of cytokine production, being outnumbered by more numerous tissue and stromal cells. Despite this, macrophages produce many of the cytokines associated with COVID-19 infection [66] and the failure of these cells to fully contain cellular damage is a likely contributor to disease outcome.

Circulating monocytes are recruited to the lungs of COVID-19 patients where monocyte attracting chemokines including CCL2 and CCL7 are increased [68]. Monocytes are classified by their expression of cell surface receptors: classical monocytes (CD14^+^CD16^−^) pass through an intermediate state (CD14^+^CD16^+^) to eventually develop into non-classical monocytes (CD14^−^CD16^+^). Increased demand for monocytes in inflammation is associated with elevated intermediate and non-classical monocytes in the blood [71] and observed in COVID-19 patients [32]. Intermediate monocytes increase in proportion with disease severity and produce granulocyte-macrophage colony-stimulating factor (GM-CSF) and IL-6. In addition, the population of large, vacuolated monocytes were observed in the peripheral blood of COVID-19 patients but not healthy individuals [72]. These cells express several macrophage markers including CD163 and CD206 in addition to CD68 and CD80 and produce a mixture of cytokines including IL-6, TNF-α and IL-10. Further studies are required to validate this subset.

In mild COVID-19, circulating monocytes express high levels of Human Leukocyte Antigen (HLA)-DR, CD11c and CD14 and are characterized by an IFN-driven transcriptional programme. In sharp contrast, patients with severe COVID-19 monocytes showed low expression of HLA-DR while expressing anti-inflammatory associated genes [19, 73, 74]. Low monocyte HLA-DR expression has been linked to monocyte anergy and associated with increased risk of secondary infections and death after sepsis [75]. Also termed monocytic myeloid-derived suppressor cells, these monocytes proliferate rapidly in the plasma of COVID-19 patients which potentially enables identification of high-risk patients [76].

SARS-CoV-2 spike protein can induce IL-8, IL-6 and TNF-α secretion in monocyte-derived macrophages (MDMs) and specifically primes COVID-19 patient-derived MDMs for IL-1β production *in vitro* [77]. IL-6 is heavily involved in the transition from innate to adaptive immune response biasing the differentiation of monocytes into macrophages, in addition to driving recruitment of neutrophils and monocytes [78]. IL-6 is also important for T cell and B cell differentiation and antibody production [79]. Macrophage produced TNF-α causes apoptosis of effector T cells [80], while IL-10 produced by lung macrophages in severe COVID-19 prevents T cell responses [81]. Cytokine production is important to elicit an effective COVID-19 immune response, however, an excess or a dysregulation of production can contribute to pathology and tissue damage. Consequently, inhibiting the function of these cytokines is predicted to provide a therapeutic benefit in COVID-19 (Table 1).

Much less data have been collected on the role of DCs in COVID-19, though studies demonstrate a decrease of plasmacytoid DCs (pDCs) in the lung and blood of patients [43, 82], which usually produce high amounts of IFN I in response to viral infection [83]. DCs’ ability to mature and activate adaptive immune cells could also be impaired in COVID-19, illustrated by reduced expression of co-stimulatory CD86 [82]. Furthermore, while it has been theorized that mast cells, basophils and eosinophils are important in COVID-19 [84, 85], reports on these cells are limited, though one study proposed that eosinophils and basophils may be important for recovery [86].

### Neutrophil extracellular traps drive COVID-19 pathology

Neutrophils serve as the first wave of immune cells recruited to infection and are prominent in viral defence and response to cellular damage [87]. A rat coronavirus infection model demonstrated that neutrophils were both involved in viral control and pathology including haemorrhagic lesions, epithelial barrier permeability and lung inflammation [88].

In COVID-19, neutrophil activation/degranulation is the largest gene signature in blood cells [89] predicting disease severity [90] and mortality [91]. An increasing number of reports have identified a high neutrophil to lymphocyte ratio to be associated with severe COVID-19 [92, 93]. Proteomic analysis has also revealed an IFN I signature in neutrophils in COVID-19 [94]. This is interesting because IFNs I were shown to be targeted by existing autoantibodies in some patients [95]. Anti-nuclear autoantibodies were also detected in COVID-19 presumably promoted by cell death or NETosis [96]. Neutrophil extracellular traps (NETs) are pyroptotically released strings of DNA/chromatin, toxic granules and fibres that are assisted by complement to capture and inactivate viruses [97]. As neutrophils express antibody binding Fc receptors, both autoantibody observations may indicate that neutrophils are forming a positive feedback loop of immune activation that leads to pathology. This overactivation could also be attributed to the phenotype of the neutrophils themselves, likely driven by persistent inflammation, elevated GM-CSF [20] and evidenced by the strange presence of hyperactive (spontaneous NET) low-density neutrophils [98] and immature neutrophils [94].

Along with activation, markers of systemic NETosis have been identified in COVID-19 patients’ blood and are linked to disease severity [99–101] (recently reviewed by Tomar *et al.* [102]. Extensive pathology may occur when large numbers of neutrophils swarm to damaged or infected tissue [63], the resulting NETosis can promote fibrosis or form a chain reaction with platelets, complement and coagulation factors leading to a thrombosis [103] that can be anti-microbial [104]. NETs are also associated with thrombotic events in COVID-19, consisting of platelets, complement (C3) and tissue factor in blood [8, 105–107]. A lower platelet count correlates with severe COVID, and may be indicative of this thrombosis [108], as are higher D-dimer levels [109, 110], which alone can predict disease outcome [111]. Thrombotic events are extremely dangerous in an intensive care unit (ICU) setting, as patients are immobile and at high risk of developing fatal clots [112–114]. Such as the NET-associated thrombotic events in the lung microvasculature, which are thought to be a key driver of lung pathology and subsequent death [99, 113, 115, 116].

Chronic kidney disease is another common outcome in COVID-19 [117]: as NETs are thought to drive kidney pathology in other diseases, they are likely to contribute to renal failure via immunothrombosis in COVID-19 [118, 119]. It is not yet clear what causes NETosis in COVID-19, though viral-induced cell death, DAMP release (e.g. Haem and ATP) and elevated cytokine levels are all implicated, especially IL-8: a potent neutrophil chemoattractant [39]. A recent study suggests that IL-6-driven complement elevation plays a role in NET formation, as activated neutrophils are known to have surfaces liable to complement binding, but also will respond to anaphylatoxins (C3a and C5a) via complement receptors [120].

## CONCLUSIONS

**Figure 1:**
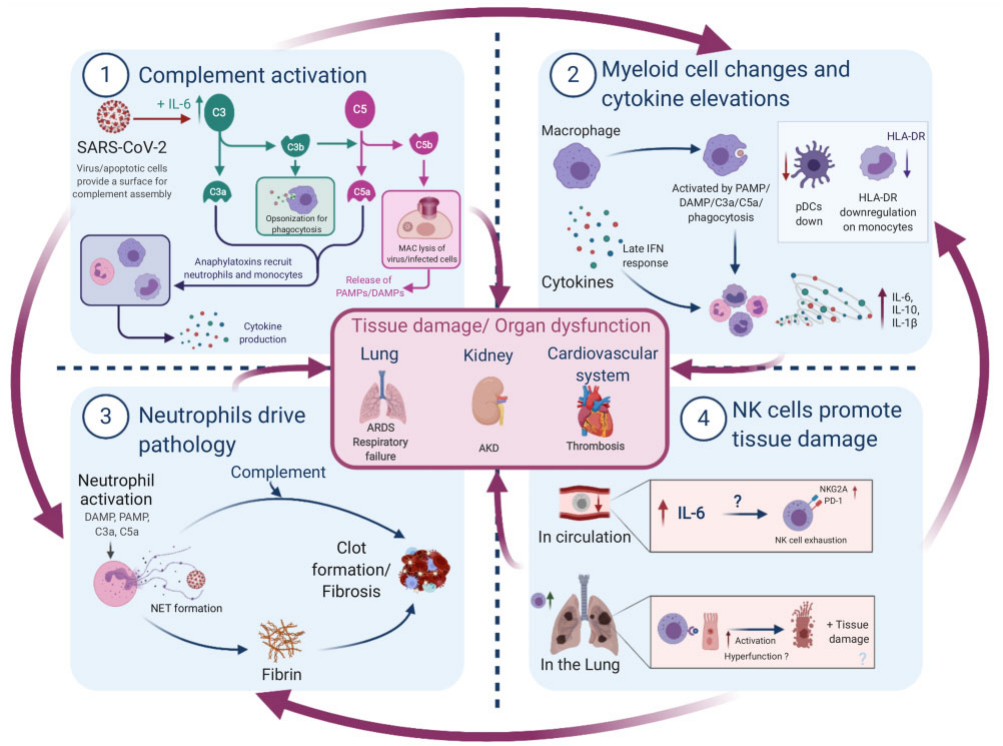
Summary of innate immune-driven pathology. Graphical summary depicting the role of innate immune components in COVID-19 pathology. (1) Complement is elevated by IL-6 and likely activated by viral/apoptotic cell membranes to initiate inflammation via anaphylatoxins. (2) Macrophages can sense tissue damage (DAMPs), complement (anaphylatoxins or C3b opsonization) or virus (PAMPs) to release cytokines; direct infection delays IFN responses. Monocytes display signs of activation or anergy through HLA-DR downregulation, while anti-viral pDCs are decreased. (3) Neutrophils respond to damage, complement and cytokines by releasing NETs consisting of fibres and DNA. NETs may act as a nucleation point for complement and coagulation factors to drive thrombosis—thought to be a major driver of mortality in COVID-19. (4) NK cells are reduced in circulation and display markers of exhaustion, which is possibly driven by elevated IL-6. These cells traffic to infected lungs where they clear virus-infected cells in mild COVID-19. However, in severe disease, these cells are possibly less effective or may contribute to tissue damage and further inflammation via DAMP release from apoptotic cells, especially if phagocytes are dysfunctional. Importantly, NK cell mechanisms are still poorly understood in COVID-19. All these innate immune factors together promote the hyperinflammation, tissue damage and organ dysfunction observed in severe COVID-19, though such inflammation may be followed by immunosuppression. AKD, acute kidney disease.

In Part 1 of this review, we concluded that SARS-CoV-2 deliberately sabotages early innate immunity, which along with inborn errors of innate sensing and IFN signalling results in increased viral load in severe COVID-19. The heavy viral burden causes cellular damage and triggers a hyperinflammatory response involving several innate immune cell types, summarized in Fig. 1. Importantly, we focus on innate immunity because there is a bias towards these cells in COVID-19, however, these findings should be taken in context with the entire immune response, as adaptive systems likely activate innate cells through antibodies and cytokines.

NK cells are principal anti-viral innate lymphocytes and display reduced circulating numbers perhaps attributed to lung recruitment in COVID-19. In severe cases, NK cells display ‘exhausted’ phenotypes and an enrichment of specialized subsets with a bias towards cytotoxicity. Without healthy phagocyte clearance of NK-targeted apoptotic host cells, such activity may result in further inflammation, however, further research is needed to evaluate the role of NK cells in COVID-19. NK cells also likely join macrophages and monocytes in contribution to the cytokine environment identified in COVID-19. There is no consensus for a cytokine storm in COVID-19. Though certain cytokines, such as high early IL-6 levels can predict the worse outcomes. In mild cases, macrophages have an early robust IFN I response, associated with viral clearance, i.e. lacking in severe patients. Additionally, low monocyte HLA-DR expression, neutrophil activation, and the ratio of neutrophils to lymphocytes have been suggested as potential identifiers of high-risk patients.

Neutrophils express an IFN I signature and produce NETs which have been implicated in immunopathology, particularly the formation of potentially fatal thrombotic events in COVID-19 patients. It is likely that activated leukocytes and NETs provide a nucleation point for the complement and coagulation pathways, and these resulting thrombi can become clogged in the small capillaries of inflamed lungs and kidneys. The heightened systemic inflammation and poor circulation in ICUs increase the likelihood of such clotting events, and thus treatments (Table 1) which target inflammation (e.g. dexamethasone), complement and coagulation are keys to reduce mortality in COVID-19.
